# Apology

**Published:** 1880-06

**Authors:** 


### Editorial.

In the April Number of this JOURNAL an article on "Placenta Prævia" written by Dr. W. W. Hipolite, was copied from the Arkansas Medical Monthly without giving the proper credit to the author or the Journal from which it was taken.

This was an unintentional failure to do justice to these parties. We are sorry the over-sight occurred, and will try to be more careful in the future.

